# Identifying characteristics of UK university students at risk of developing adverse markers of health and related behaviours across one year at university: a latent transition approach

**DOI:** 10.1186/s12889-025-21759-8

**Published:** 2025-03-18

**Authors:** Matthew J. Savage, Laura C. Healy, Eleanor L. Procter, Philip J. Hennis, Ruth M. James

**Affiliations:** 1 Diabetes Research Centre, College of Life Sciences, University of Leicester, Leicester, UK; 2 SHAPE Research Group, School of Science and Technology, Nottingham Trent University, Nottingham, UK

**Keywords:** Young adults, Lifestyle, Gender, Ethnicity, Well-being

## Abstract

**Introduction:**

University students are a population notorious for developing adverse markers of health and related behaviours that can have negative consequences for current and future health status. However, there is a dearth of literature devoted to identifying students at greater risk of developing poorer health-related outcomes. The current study aimed to identify characteristics of UK university students at risk of developing adverse markers of health and related behaviours across one year at university.

**Methods:**

Four hundred and thirty-eight students completed an online self-report survey to assess markers of health and related behaviours in term one (October) and term three (April) in one of three academic years (2021–22, 2022–23, and 2023–24). Latent Profile Transition analysis was employed to generate health-related profiles and assess transitions over time.

**Results:**

Four latent profiles were detected, largely influenced by physical activity behaviours and psychological markers. The majority of students were identified in profiles considered as less healthy and remained in those profiles over time. Women and trans and gender diverse (TGD) students, and students in their second year at university were at greatest risk of being in, and remaining in, less healthful profiles.

**Conclusions:**

Most students identify and remain in less healthful profiles throughout the academic year. Students that transition between profiles are more likely to transition to less healthful profiles. Work to develop bespoke interventions aimed at students with higher-risk demographic characteristics should now be prioritised.

## Introduction

Universities offer a distinctive environment where students face new social, cultural, economic, and environmental circumstances. Attending university is often linked with participating in social events centred around drinking alcohol [1], financial struggles [2], and external pressures from family and peers [3, 4], which can adversely affect health and lifestyle indicators [5–7]. Indeed, literature has demonstrated that university students develop poor health-related habits and behaviours [8, 9] with substantial proportions engaging in sub-optimal movement behaviours [6, 9, 10], binge drinking [11], and poor dietary practices [6, 9]. Participating in these behaviours may have negative implications for cardiometabolic health with evidence suggesting that students experience significant increases in body mass (over 0.5kg) [12], waist circumference (1.9cm) [13], and fat mass (0.8kg) [14] across the timeline of a degree programme. Worryingly, the development of adverse cardiometabolic health outcomes during early adulthood has been associated with a heightened risk of developing physical morbidities later in life, such as type 2 diabetes and hypertension [15], which could exacerbate the strain on overstretched public health systems throughout the UK [16].

To resolve this issue, previous research has focused on identifying interventions to improve students' exercise and dietary practices, with varying success [17]. However, these variable-centred approaches often fail to consider nuanced characteristics such as gender and ethnicity, and do not consider how health-related behaviours may cluster, hindering the effectiveness of interventions aimed at the general student population. Recent literature has highlighted differences in health outcomes and related behaviours among subpopulations of students (e.g., genders and ethnic backgrounds) of different genders and ethnicities, as well as across different years of study [9, 18, 19]. It is therefore pertinent that study designs consider these factors and employ person-centred approaches to identify characteristics of students at greatest risk of developing negative health and behavioural outcomes whilst attending university.

Person-centred analyses enable researchers to generate profiles based on variables of interest, understand how individuals transition between these profiles, identify those at risk of transitioning to negative profiles, and examine how these transitions relate to key variables [20]. To date, few studies have utilised this approach and have largely focused on generating clusters related to substance use (i.e., drug and alcohol use, and tobacco smoking) [21–23] and sexual behaviours in university students [24]. This means important lifestyle behaviours (i.e., movement and dietary habits) that are notoriously problematic in students were not considered [6, 9]. Additionally, these studies were conducted outside of the UK and results may therefore not reflect specific cultural, social, and educational factors (including social opportunities, behavioural habits, and methods of tuition) unique to UK higher education [25–28].

Only a single UK study has employed Latent Transition Analysis (LTA) to identify the pattern of change between health-related profiles across the duration of a typical undergraduate degree programme (3-years) using a broader spectrum of health-related lifestyle outcomes (i.e., smoking, alcohol intake, fruit and vegetable consumption, and vigorous physical activity levels) [29]. The study identified three classes (higher risk; moderate risk, active; & moderate risk, inactive) and indicated that students generally (87%) transitioned to similar or the same classes over the duration of their studies [29]. The study also found that being a White male or a female of Black, Mixed, or Other ethnicity was associated with a higher likelihood of being in a 'higher risk' class [29]. However, the overall study sample size and the proportion of male and Minoritised Ethnicity students was small (*n* = 128). As such, it is unlikely that results can be generalised to that of the wider UK student population, particularly given the increased diversity within the student population in recent years [30].

Furthermore, data within the study was collected at a single time point in the first term (September to December) of three academic years and as such, the latent classes generated and observed transitions may discount important fluctuations in health-related behaviours within an academic year [29]. Accurately capturing transitions during this period would enable stakeholders to target health-based support and initiatives at students who would benefit most. The current study therefore aimed to 1) identify latent health-related profiles in UK university students at the beginning and the end of an academic year, 2) examine how university students transition between health-related profiles across an academic year, and 3) identify characteristics of students at risk of transitioning into negative health-related profiles across an academic year. It was hypothesised that students who are White men or women of Minoritised Ethnicity would be more likely to transition to poorer health-related profiles over time.

## Methods

### Study design and participants

**Fig. 1:**
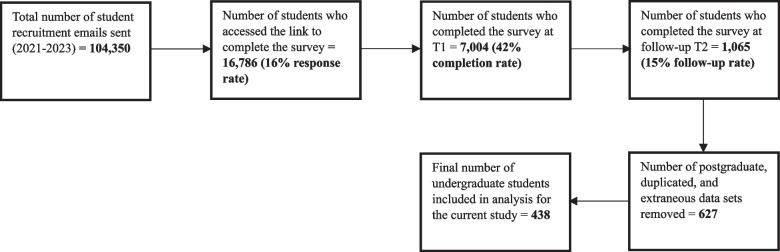
Recruitment data for participation in the survey (2021–2023)

Students were recruited to participate in the Student Health Study, a longitudinal research project examining the health and overall wellness of university students in the East Midlands region of the UK. 438 students completed an online self-report survey in term one (October) and term three (April) in one of three academic years (2021–22 *n* = 144, 2022–23 *n* = 113, and 2023–24 *n* = 181). Detailed participant recruitment information is provided in Fig. 1. The investigation adhered to the STROBE guidelines [31]. All participants provided informed consent prior to joining the study, and ethical approval was granted by the School of Science and Technology Human Ethics Committee at Nottingham Trent University (application ID: 19/20–76).

### Survey design

The survey included socio-demographic questions (8 items) and a health history question (1 item: "Do you have any diagnosed long-term health condition(s)?"). Additionally, the questionnaire featured two validated scales: Cohen’s Perceived Stress Scale (PSS) [32] and the Short Warwick-Edinburgh Mental Wellbeing Scale (S-WEMWBS). The PSS uses a 5-point Likert scale (0 = 'Never' to 4 = 'Very often'), with total scores ranging from 0 to 40, where higher scores indicate higher levels of perceived stress. The S-WEMWBS also uses a 5-point Likert scale (1 = 'None of the time' to 5 = 'All of the time'), with total scores ranging from 7 to 35, where higher scores signify better mental wellbeing. Both scales have been validated in UK students (Cronbach's alpha = 0.89 and Composite reliability (ρc) = 0.88, respectively) [33, 34].

The survey also included the International Physical Activity Questionnaire – Short Form (IPAQ-SF) to assess moderate (MPA), vigorous (VPA), and walking (WPA) intensity physical activity, as well as time spent sitting on weekdays over the past seven days. Responses were scored following the IPAQ protocol (www.ipaq.ki.se) to determine the amount of MVPA performed per week, a measure validated in university students [35, 36].

Additionally, the United States Alcohol Use Disorders Identification Test – Consumption (USAUDIT-C) was used to identify risky drinking behaviour. The USAUDIT-C is a 3-item scale, each item is scored on a 6-point Likert scale (0 = 'Never' or '1 drink' to 5 = 'Daily' or '10 or more drinks') with total scores ranging from 0 to 18. Scores of ≥ 7 for women and ≥ 8 for men indicate a positive risk for alcohol use disorders. The USAUDIT-C has been validated in university students [37].

The survey also featured a single-item Sleep Quality Scale (SQS) [38] and a Short Form Food Frequency Questionnaire (SFFFQ) [39]. The SQS assesses subjective night-time sleep quality over the past seven days using a 10-point Likert scale, with 0 indicating 'terrible' and 10 indicating 'excellent'. The SFFFQ consists of 27 items where participants reported the frequency of their consumption of each item during a typical week, selecting from eight frequency categories ranging from 'rarely or never' to 'five or more times per day'. A diet quality score (DQS) was calculated based on the SFFFQ protocol [39]. Both scales have been previously validated [38, 39].

### Statistical analysis

Initially, Latent Profile Analysis (LPA) was conducted to identify health and related profiles at each timepoint. A series of models, featuring an increasing number of profiles from 2 to 5, were analysed to ascertain whether more complex (i.e., more profiles) or simpler (i.e., fewer profiles) models best described the data. These models were estimated using data from both time points, with time points treated as independent of one another. The current study determined the most appropriate model using several criteria in accordance with recommendations [40] and consistent with previous literature [41, 42]. Model-fit indices including the Akaike Information Criterion (AIC), the Bayesian Information Criterion (BIC), and the sample-adjusted Bayesian Information Criterion (aBIC) were used, with lower values indicating better model fit [43, 44]. Furthermore, model entropy and class membership probabilities were evaluated to provide further detail surrounding model selection. Theoretical and practical interpretations of the final profiles in relation to item response probabilities were also considered to aid in determining the most meaningful model with the smallest number of profiles.

Once the optimum number of profiles had been determined, Latent Transition Analysis (LTA) was conducted to assess movement between profiles over time. Measurement invariance was assessed using multiple models with increasing constraints (Configural (unconstrained) model, equal means across groups, equal means and variances across groups, & equal means, variances, and class sizes across groups). To determine the most appropriate model, model fit indices (AIC, BIC, & aBIC) and the Likelihood Ratio Difference Test (LRDT) were compared between models. Descriptive statistics (i.e., percentages) were used to highlight proportions within covariate categories that transitioned between profiles. For these analyses, gender was grouped into three categories: men, women, and TGD (Trans and Gender Diverse; those who experience incongruence between their sex assigned at birth and gender identity) [45]. Ethnicity was categorised into two groups: White and Minoritised ethnicity (ME). All analyses were conducted in MPlus (version 7, Muthen & Muthen).

## Results

Participant information is provided in Table 1. Model fit indices for 2–5 latent profiles across both time points are presented in Table 2. Whilst fit indices (aBIC, BIC & AIC) were lowest for 5-profiles at each time point, at least one profile had a very low probability of membership (< 5%) raising concerns about overfitting and generalisability [46]. Such small profiles may present reflect random noise rather than meaningful subgroups, leading to unreliable conclusions and practical irrelevance and thus, should be avoided [47, 48]. The next lowest fit indices belonged to the 4-profile solution, which also had a higher entropy and better assignment probabilities at T1 compared to the 5-profile solution. Additionally, sample distribution was better at both time points for the 4-profile solution. The 4-profile solution was therefore deemed most appropriate based on model fit indices and interpretability of the model. The 4-profile LTA model was ran with different levels of measurement invariance and constraints to determine the most appropriate solution [49] (Table 3). The model with equal means and variances showed the lowest BIC, indicating the best fit, while an entropy value > 0.8 further supported its strong overall performance [50]. Additionally, the LRDT was < 0.05, suggesting this model was not worse than models with less constraints. As such, this model was selected as most appropriate as the final LTA model.

**Table 1:** Participant characteristics presented as n (%)

**Age (years)**	Total (*n* = 438)	21–22 (*n* = 144)	22–23 (*n* = 113)	23–24 (*n* = 181)
18	82 (18.7)	19 (13.2)	17 (15.0)	46 (25.4)
19	105 (24.0)	23 (16.0)	32 (28.3)	50 (27.6)
20	93 (21.2)	37 (25.7)	24 (21.2)	32 (17.7)
21	66 (15.1)	27 (18.8)	19 (16.8)	20 (11.0)
22–25	44 (10.0)	23 (16.0)	9 (8.0)	12 (6.6)
26–35	25 (5.7)	9 (6.3)	9 (8.0)	7 (3.9)
35 +	23 (5.3)	6 (4.2)	3 (2.7)	14 (7.7)
Not specified	0 (0.0)	0 (0)	0 (0)	0 (0)
**Gender**				
Women	295 (67.4)	95 (66.0)	81 (71.7)	119 (65.7)
Men	116 (26.5)	35 (24.3)	25 (22.1)	56 (30.9)
TGD	26 (5.9)	14 (9.7)	7 (6.2)	5 (2.8)
Not specified	1 (0.2)	0 (0)	0 (0)	1 (0.6)
**Ethnicity**				
White	353 (80.6)	129 (89.6)	95 (84.8)	129 (71.3)
ME	83 (18.9)	15 (10.4)	17 (15.2)	51 (28.2)
Not specified	2 (0.5)	0 (0)	1 (0.9)	1 (0.6)
**Year of study**				
1st Year	170 (38.8)	44 (30.6	34 (30.1)	92 (50.8)
2nd Year	142 (32.4)	46 (31.9)	41 (36.3)	55 (30.4)
3rd Year	126 (28.8)	54 (37.5)	38 (33.6)	34 (18.8)

**Table 2:** Model fit indices for latent profile analysis containing 2–5 profiles at both time points

**Time Point 1**					
# of classes	aBIC	BIC	AIC	Entropy	Assignment Probabilities
2	23,522.75	23,643.35	23,488.22	0.71	0.91—0.92
3	23,438.20	23,606.40	23,390.04	0.80	0.78—0.92
4	23,356.16	23,571.96	23,294.37	0.82	0.85—0.92
5	23,289.40	23,552.80	23,213.98	0.79	0.65—0.92
**Time Point 2**					
# of classes	aBIC	BIC	AIC	Entropy	Assignment Probabilities
2	24,655.44	24,776.03	24,620.91	0.75	0.90—0.94
3	24,524.68	24,692.87	24,476.52	0.77	0.77—0.91
4	24,453.26	24,669.06	24,391.47	0.80	0.73—0.92
5	24,396.65	24,660.05	24,321.23	0.81	0.79—0.92

**Table 3:** Model fit indices for 4-profile LPTA with varying levels of measurement and variance constraints

Model	aBIC	BIC	AIC	Entropy	Loglikelihood	LRDT *p* value
Configural (unconstrained) model	44,918.00	46,361.94	44,504.54	0.91	−21,797.27	
Equal means across groups	45,035.45	45,679.67	44,850.98	0.86	−22,222.49	1.00
Equal means and variances across groups	44,987.55	45,517.53	44,835.80	0.86	−22,250.90	1.00
Equal means, variances, and class sizes across groups	45,100.11	45,745.10	45,243.69	0.78	−22,392.06	1.00

**Fig. 2:**
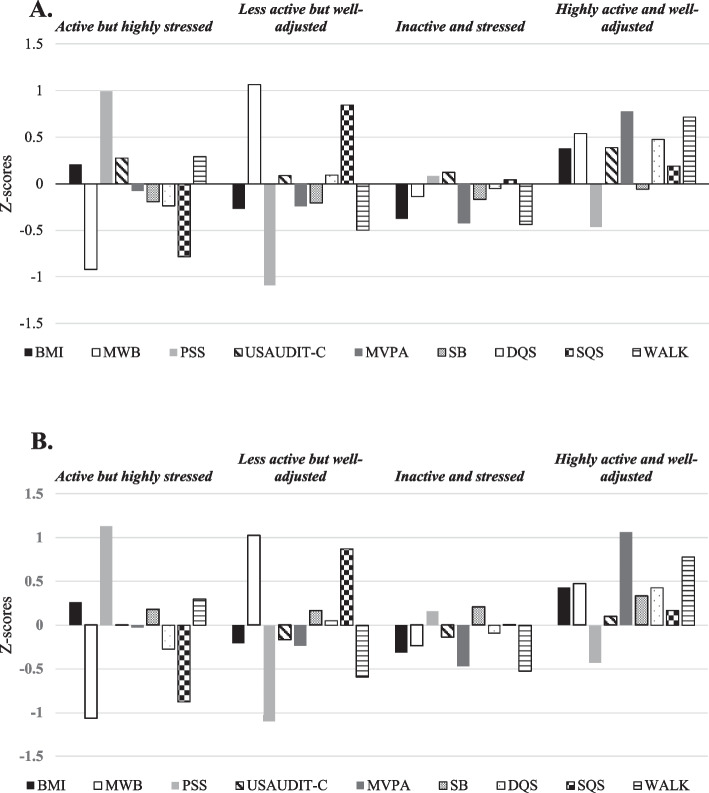
Graphical illustration of behavioural profiles for the 4-profile model at T1 (**A**) and T2 (**B**)

**Fig. 3:**
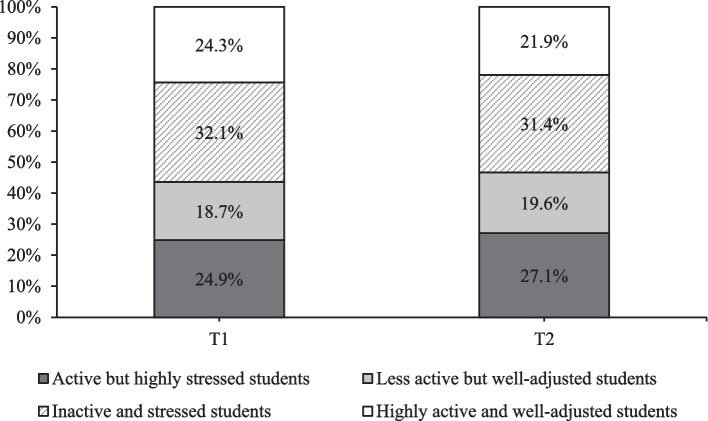
The probabilities of being assigned to each profile at both time points

The four profiles denoted by the LTA model are represented in Fig. 2A (T1) & B (T2), and the probability of membership to each profile at both time points is presented in Fig. 3. Profiles were interpreted using z-scores in relation to the sample mean. Z-scores were denoted using similar approaches to previous literature with values of ± 1 SD classified as very high/low, ± 0.5 to 1 SD as high/low, and −0.5 to + 0.5 (including 0) as above/below average [49]. Mean ± SD values for all variables in each individual profile are displayed in Table 4. The four profiles were labelled as follows:

1. Active and highly stressed**—**These students experience low mental well-being and high perceived stress, with poor sleep quality and diet. However, they maintain an active lifestyle with walking levels greater than the sample mean, and with physical activity levels being very close to the profile mean. These students also have a BMI slightly above the sample mean. The label emphasises their stress and mental health challenges despite some active habits.
2. Less active and well-adjusted—Students in this group have high mental well-being, low perceived stress, and good sleep quality, but they engage in less physical activity (low walking and MVPA). Their sedentary behaviour and diet quality are above the sample mean whereas their BMI is below the sample mean.
3. Inactive and stressed—This profile represents students with below-average physical activity, diet quality and mental well-being, and above-average perceived stress, and sedentary behaviour.
4. Highly active and well-adjusted—These students are characterised by very high levels of physical activity (MVPA and walking), above-average mental well-being, diet quality and sleep. They also have below-average perceived stress.

**Table 4:** Means and SD for variables in all profiles

	Profile 1 (active and highly stressed students)		Profile 2 (less active but well-adjusted students)		Profile 3 (inactive and stressed students)		Profile 4 (highly active and well-adjusted students)	
Variable	*Mean*	*SD*	*Mean*	*SD*	*Mean*	*SD*	*Mean*	*SD*
BMI (kg/m2)	25.4	9.7	22.8	5.0	22.3	4.9	26.4	10.3
MWB	16.9	2.6	24.4	3.1	19.9	3.5	22.4	3.5
PSS	28.4	5.4	13.0	6. 0	21.7	7.0	17.7	6.6
USAUDIT-C	6.3	3.4	5.8	3.6	5.9	5.8	6.7	4.5
MVPA (mins/week)	362	452	266	249	158	520	865	891
SB (hours/week)	36.4	30.2	36.1	21.3	37.1	31.9	39.8	32.6
DQS	9.4	1.8	10.0	2.6	9.7	2.0	10.7	2.9
SQS	3.8	2.3	7.5	1.9	5.7	2.2	6.0	2.0
WALK (mins/week)	591	504	234	182	262	199	783	566

Profiles remained relatively stable, although increases in USAUDIT-C scores and decreases in SB were found across all profiles over time (Fig. 3A & B).

Figure 3 shows the probability of profile classification at both time points. Generally, the proportions of participants in each profile were stable over time. At each timepoint, ‘Inactive and stressed’ contained the highest proportion of students (32.1% & 31.4% respectively), and ‘Less active but well-adjusted’ had the lowest (18.7% & 19.6% respectively).

**Fig. 4:**
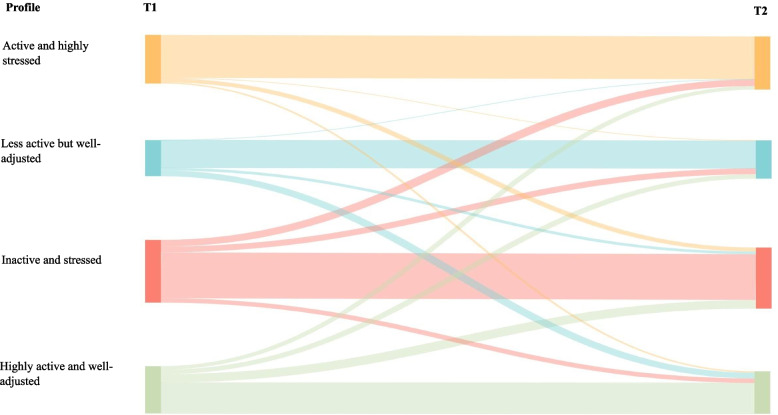
Transitions between profiles across time points

Figure 4 displays the proportion of students transitioning between profiles over time based on transition probabilities. When transitioning between T1 and T2, the majority of students remained in their original profile (75.3%), most commonly, ‘Inactive and stressed’ (23.5%) followed by ‘Active but highly stressed’ (21.8%). Of those who transitioned to a new profile (*n* = 107), students were most likely to transition from ‘Inactive and stressed’ (35.5%) and least likely to transition from ‘Active but highly stressed’ (12.1%). Equally, students who transitioned to a new profile were most likely to transition to ‘Inactive and stressed’ (31.8%) and least likely to transition to ‘Active but highly stressed’ (21.5%). The most common transition pattern for those who transitioned to a new profile was from ‘Highly active and well-adjusted’ to ‘Inactive and stressed (17.8%). Over half (58.4%) of all students experienced transitions into more negative profiles (i.e., remained in or transitioned to ‘Active but highly stressed’ or ‘Inactive and stressed’).

The proportions of students within each profile at both time points based on gender, ethnicity, and year at university are presented in Table 5. ‘Active and highly stressed’ had the highest proportion of women across time points, with membership ranging from 54.7% to 77.1%. Equally, ‘Active and highly stressed’ had the greatest proportion of TGD students at both time points, with membership ranging from 1.2% to 12.8%. The proportion of men was greatest in ‘Less active but well-adjusted’ at T1 but was highest in ‘Highly active and well-adjusted’ at T2. The proportion of men within all profiles ranged from 10.2% to 43.2%. The highest proportion of Minoritised ethnicity students was in ‘Less active but well-adjusted’ across time points with membership ranging from 14.7% to 22.1%. Conversely, ‘Active and highly stressed’ had the highest proportion of White students at T1 whereas both ‘Active and highly stressed’ and ‘Inactive and stressed’ had the highest proportion of White students at T2 with membership ranging from 77.9% to 85.3%. The highest proportion of students in year 1 was in ‘Less active but well-adjusted’ at T1 but ‘Highly active and well adjusted’ at T2 with membership ranging from 26.7% to 57.9%. ‘Active and highly stressed’ had the highest proportion of year 2 students across time points with proportions ranging from 22.2% to 47.7%. ‘Less active but well-adjusted’ and ‘Inactive and stressed’ had the highest proportion of year 3 students at T1, and ‘Less active but well-adjusted’ also had the highest proportion of year 3 students at T2 with membership ranging from 20.0% to 40.7%.

**Table 5:** Proportion of covariate categories in each profile across time points (%)

**Time Point 1**	**Profile**			
Covariate	*Active and highly stressed*	*Less active and well-adjusted*	*Inactive and stressed*	*Highly active and well-adjusted*
**Gender**				
* Women*	77.1	54.3	74.5	58.5
* Men*	10.1	43.2	20.6	38.7
* TGD*	12.8	2.5	5.0	2.8
**Ethnicity**				
* White*	85.3	79.0	79.4	82.1
* ME*	14.7	21.0	20.6	17.9
**Year at university**				
* 1*	30.3	44.4	42.6	38.7
* 2*	47.7	22.2	24.1	34.9
* 3*	22.0	33.3	33.3	26.4
**Time Point 2**	**Profile**			
Covariate	*Active and highly stressed*	*Less active and well-adjusted*	*Inactive and stressed*	*Highly active and well-adjusted*
**Gender**				
* Women*	76.5	61.6	75.6	54.7
* Men*	12.6	37.2	21.4	43.2
* TGD*	10.9	1.2	7.6	2.1
**Ethnicity**				
* White*	84.0	77.9	84.0	83.2
* ME*	16.0	22.1	20.6	16.8
**Year at university**				
* 1*	32.8	26.7	40.5	57.9
* 2*	42.0	31.4	32.1	23.2
* 3*	25.2	40.7	32.1	20.0

The proportions of covariate categories within transition patterns are presented in Table 6. Of those who transitioned into more negative profiles (i.e., remained in or transitioned to ‘Active and highly stressed’ or ‘Inactive and stressed’), the majority were women, White, and in 1st or 2nd year at university (Table 6). When expressed as a proportion of the number of students within each covariate category, the vast majority of TGD students (88.5%) transitioned in this manner, as did most women (64.4%) (Table 7). Equally, most White (59.5%) and ME students (55.4%) transitioned in this way as did students in first (54.1%), second (64.8%), and third year students (57.1%) (Table 7).

**Table 6:** Proportion of covariate categories within each transition pattern

Transition pattern (profile number)	Covariate (%)							
	*Gender*				*Ethnicity*	*Year at university*		
	Female	Male	TGD	White	Minoritised ethnicity	1	2	3
‘Active and highly stressed’ to ‘Active and highly stressed’	80.7	11.6	7.7	85.0	15.0	31.0	48.3	20.7
‘Active and highly stressed to ‘Less active but well-adjusted’	100.0	0.0	0.0	0.0	100.0	0.0	0.0	100.0
‘Active and highly stressed to ‘Inactive and stressed’	26.2	0.0	73.8	100.0	0.0	10.0	53.6	36.4
‘Active and highly stressed to ‘Highly active and well-adjusted’	100.0	0.0	0.0	67.2	32.8	64.2	35.8	0.0
‘Less active but well-adjusted’ to ‘Active and highly stressed’	100.0	0.0	0.0	100.0	0.0	0.0	0.0	100.0
‘Less active but well-adjusted’ to ‘Less active but well adjusted’	61.3	38.7	0.0	88.3	11.7	31.1	27.1	41.8
‘Less active but well-adjusted’ to ‘Inactive and stressed’	100.0	0.0	0.0	19.8	80.2	100.0	0.0	0.0
‘Less active but well-adjusted’ to ‘Highly active and well-adjusted’	0.0	85.8	14.2	59.0	41.0	82.1	9.2	8.7
‘Inactive and stressed’ to ‘Active and highly stressed’	55.9	5.5	38.6	100.0	0.0	52.0	0.0	47.9
‘Inactive and stressed’ to ‘Less active but well-adjusted’	71.5	20.6	7.9	69.1	30.9	21.3	33.9	44.8
‘Inactive and stressed’ to ‘Inactive and stressed’	77.5	22.3	0.2	80.1	19.9	37.6	29.4	33.0
‘Inactive and stressed’ to ‘Highly active and well-adjusted’	80.2	19.8	0.0	46.8	53.2	100.0	0.0	0.0
‘Highly active and well-adjusted’ to ‘Active and highly stressed’	58.9	41.1	0.0	39.5	60.5	12.3	51.9	35.8
‘Highly active and well-adjusted’ to ‘Less active but well-adjusted’	49.3	50.7	0.0	30.3	69.7	13.5	63.2	23.3
‘Highly active and well-adjusted’ to ‘Inactive and stressed’	55.6	27.0	17.4	87.4	12.6	38.4	35.9	25.7
‘Highly active and well-adjusted’ to ‘Highly active and well-adjusted’	59.9	40.1	0.0	92.6	7.4	45.7	28.4	25.8

**Table 7:** Proportions of covariate categories that transition into less healthful profiles (‘Active and highly stressed’ & ‘Inactive and stressed’) expressed as a proportion of the population that transition into negative health-related profiles (*n* = 256) and as a proportion of the number of students within each covariate category in the total population (*n* = 438)

Covariate categories			
Gender	*n*	Proportion of the population transitioning negatively (*n* = 256) (%)	Proportion of covariate categories within entire population (*n* = 438) (%)
Women	190	74.2	64.4
Men	43	16.8	37.1
TGD	23	9.0	88.5
**Ethincity**			
White	210	82.0	59.5
Minoritised ethnicity	46	18.0	55.4
**Year of study**			
1st Year	92	35.9	54.1
2nd Year	92	35.9	64.8
3rd Year	72	28.1	57.1

## Discussion

The aims of the current study were three-fold; 1) to identify latent health-related profiles in UK university students at the beginning and the end of an academic year, 2) to examine how university students transition between health-related profiles across an academic year, and 3) to identify characteristics of students at risk of transitioning into negative health-related profiles across an academic year. The findings indicate the presence of four distinct health-related profiles that largely remain stable across an academic year. Additionally, these data show that 57% of students remain within one profile over time. Further, it is noteworthy that TGD and 2nd year students may be at greatest risk of transitioning into more negative health profiles throughout the course of an academic year.

### Discussion of previous literature

Previously, few studies have used longitudinal data to assess alterations in clusters of health-related markers and behaviours in university students [21–24]. These studies largely focus on substance abuse and take place outside of the UK [21–24] meaning information surrounding the clustering of health-related markers and behaviours in UK university students is limited. To date, only one study has utilised a person-centred approach to examine the classification of health-related markers and behaviours in UK university students [29]. The findings identified three classes and indicated that the vast majority of students had multiple adverse lifestyle-related risk factors (i.e., were not adhering to guidelines), meaning large proportions were identified in more negative ‘at risk’ classes [29]. However, the study used categorical variables to identify classes of students that were at different levels of ‘risk’ based on UK guidelines [29]. Whilst informative, categorical data can suppress nuanced information about patterns of means and variances in variables that can be more accurately captured by continuous data (i.e., z-scores). The use of continuous data in the current study therefore provides further in-depth knowledge and understanding of health-related profiles in university students.

### Latent profile analysis

The four profiles identified in the current study were distinctly different from one another and were largely characterised by alterations in physical activity behaviours (MVPA & walking) and psychological outcomes (MWB, PSS). However, it is also noteworthy that BMI, sleep, and diet quality also had a considerable influence on profile classification. Specifically, ‘Active and highly stressed’ and ‘Less active and well-adjusted’ were largely determined by psychological markers in addition to walking and sleep. In contrast, ‘Inactive and stressed’ was characterised by variables predominantly centred around the population mean, and ‘Highly active and well-adjusted’ was heavily influenced by physical activity behaviours, psychological markers and diet quality.

When assessing the proportions of students within each profile across time points, most students (57%−58.5%) were identified to profiles that were associated with more negative health-related markers and behaviours (‘Active and highly stressed’; 24.9%−27.1% or ‘Inactive and stressed’; 31.4%−32.1%), characterised by lower levels of physical activity, poorer psychological markers and worse lifestyle habits (i.e., sleep and diet quality). This is in accordance with an abundance of previous literature suggesting that university students are generally an unhealthy population characterised by low levels of physical activity, poor mental health, sub-optimal dietary practices, and hazardous alcohol drinking behaviours [6, 10, 11, 51, 52].

However, a considerable proportion of students (43%−41.5%) were classified into more healthful profiles (‘Less active and well-adjusted’; 18.7%−19.6% or ‘Highly active and well-adjusted’;24.3%−21.9%) characterised by higher levels of physical activity, better psychological markers, and more favourable lifestyle habits (i.e., sleep and diet quality). This is a substantially larger proportion of the student population than suggested in previous literature [29] and provides novel insight into the current context of university students health. This unexpected finding may result from a societal shift towards increased health consciousness in young adults in comparison to that of previous generations. Indeed, larger proportions are beginning to take ownership of their health through increasing physical activity, improving dietary practices, and abstaining from smoking [53]. This may also be true of current university students, of whom, a 2023 survey characterised as ‘generation sensible’ as data suggests they are attempting to save money through reducing expenditure on social activities and alcohol [54]. This also may be driven by increased efforts to improve health awareness during the COVID-19 pandemic [78], meaning young adults may now engage in healthier behaviours to reduce the risk of developing harmful symptoms associated with transmissible virus’s such as COVID. Nonetheless, as society becomes more health aware, it is plausible that a growing proportion of university students are beginning to prioritise health and wellbeing over partaking in traditional student practices that may be hazardous to health, such as binge drinking.

As such, distinctly different groups are emerging that are at opposite ends of the spectrum in relation to prioritising health and health-based practices. It may therefore no longer be necessary to develop health-based initiatives for university students as a general population and instead, targeting individuals within less healthful profiles may be a more beneficial approach.

### Latent profile transition analysis

This is further evidenced when exploring transitions over time. The present study showed that 75.3% of students remained in their original profiles, suggesting high levels of stability and indicating that most students do not substantially alter their behavioural patterns over the duration of one year at university. This is in line with previous literature in the area that uses a similar analytical approach [21, 22, 24, 29]. However, when examining all transitions over time (stable & un-stable), over half of the students included in the current study remained in or transitioned into less healthful profiles (‘Active and highly stressed’ or ‘Inactive and stressed’). Furthermore, of those who transitioned to a new profile, the majority transitioned to a less healthful profile, and the most prevalent transition pattern (outside of the stable transitions) was from the profile considered as most healthful (‘Highly active and well-adjusted’) to the profile considered as least healthful (‘Inactive and stressed’). These findings are in accordance with previous literature indicating that periods of change (i.e., from secondary to higher education) can be synonymous with the adoption of poorer health-related behavioural patterns [55], and that students who transition between behaviour-related profiles adopt riskier substance use patterns across an academic year [21, 24]. Taken together, these data demonstrate that large proportions of students begin the academic year with adverse markers of health and related behaviours that are sustained throughout the year. Additionally, albeit to a lesser extent, there are a number of students that begin the year in more positive health-related profiles and transition to more negative profiles over the duration of the academic year. As such, further exploration of these students is required in order to begin addressing this issue through developing targeted health-based initiatives for those who may benefit most.

### Gender, ethnicity and year at university

The current study indicates that high proportions of TGD students (88.5%) and students who are women (64.4%) remain in or transition to negative health profiles across an academic year in comparison to students who are men (37.1%). This is in line with the limited previous literature that has indicated that TGD students are at greater risk of developing poorer health-related outcomes in comparison to their cis-gendered peers, particularly related to movement behaviours and markers of mental health [56–58]. Additionally, women have been shown to be less physically active and have poorer outcomes related to psychological wellbeing than men [6, 59–61].

Interestingly, similar proportions of White (59.5%) and Minoritised Ethnicity (55.4%) students remained in or transitioned to negative profiles. The lack of disparity between White and Minoritised Ethnicity students may be somewhat surprising given previous data has shown those of Minoritised Ethnicity are disadvantaged in relation to accessing mental health services [62] and opportunities to engage in physical activity [63–65]. These findings may therefore reflect positive work that has been completed to reduce inequalities in the health sector [62, 66].

Furthermore, a higher proportion of students in second year (64.8%) remained in or transitioned into negative profiles in comparison to students in first (54.1%) or third year (57.1%). Whilst literature examining differences between year groups is limited, longitudinal data suggests students' health and behaviours become worse throughout the duration of an undergraduate degree [67]. Additionally, some literature suggests that anxiety is highest in the second year of university and students could benefit from further support during this period [68].

In addition to demographic characteristics such as gender, ethnicity, and year of study, it is also important to note that a plethora of other sociodemographic and economic factors likely influence these transition patterns. Previously, literature has demonstrated that students from lower socioeconomic backgrounds have poorer dietary habits [69] and spend less time actively commuting to university compared to those from non-low socio-economic backgrounds [70]. However, exploring these factors in detail is outside the scope of the current study. As such, further investigation is required to understand the extent to which a multitude of sociodemographic and economic factors influence students transitions between health-related profiles whilst at university.

Taken together, these data indicate that students identifying as TGD or women and are in their second year at university are at greatest risk of presenting with and transitioning to more negative health-related profiles across an academic year. As well as health and wellbeing, these findings could also have important implications for academic achievement. Indeed, literature has demonstrated that developing less healthful behaviours and outcomes (e.g., inadequate physical activity and increased stress) is associated with impaired cognitive function [71, 72] and poorer academic grades [73, 74]. As such, university stakeholders should consider targeting students within ‘at-risk’ demographics (i.e., women and TGD students, and students in second year) when developing health-based initiatives as a mechanism to enhance health and wellbeing, and subsequently, academic performance in the future. It should be noted that some students outside of these ‘at risk’ demographics were also identified in and transitioned to less healthful profiles. Higher education institutions should therefore explore strategies to ensure students who are initially identified in more healthful profiles remain in these profiles throughout the academic year, while supporting students in less healthful profiles to transition in a positive manner.

### Strengths and limitations

The novel statistical approach employed in the current study has enabled the identification of distinct health-related profiles in university students and has provided crucial information surrounding those at greater risk of remaining in or transitioning to negative profiles over the course of an academic year. This builds upon previous work in the area through the inclusion of a relatively large, longitudinal sample size, that incorporates large proportions of demographics previously underrepresented in research within this area, meaning it is more likely that results can be generalised to wider student populations [21–24, 29]. This is further evidenced through the relative comparability of the current study’s sample demographics to that of the wider UK student population (57% female & 72% White) [30]. Additionally, the use of a broad range of health-related outcomes and behaviours facilitated a holistic approach that is needed to begin addressing the broader issue of university student health. The current study therefore offers valuable insights that can inform targeted interventions and policies aimed at improving the overall well-being of university students.

Despite the clear strengths of the current study, some limitations and directions for future research should be acknowledged. The study design only enabled assessment of transitions within one year at university. Typically, an undergraduate degree takes place over the duration of three years. As such, it is possible that students within the present sample altered behavioural patterns outside of the participation window. Whilst previous research has attempted to capture fluctuations across a three-year degree programme, the longitudinal sample size incorporated was too small generate results that are representative of the wider student population. It is therefore vital that future research aims to incorporate large sample sizes across the entirety of an undergraduate degree and employs a person-centred approach to statistical analysis to further enhance knowledge surrounding the influence of university life on students’ health. The use of objective measures of health and behaviours would also advance knowledge on this topic further. Currently, all literature using similar study designs and analytical approaches relies on self-report data [21–24, 29] which may lead to inaccuracies when reporting lifestyle behaviours including overestimating physical activity levels and underestimating alcohol consumption and sedentary behaviour [75–77]. However, using validated questionnaires and survey items reduced the impact of these inaccuracies. Additionally, the use of objective measures of health (i.e., anthropometric measures, blood markers etc.) would be valuable in gaining further understanding the impact of university life on overall health status. As such, research should aim to incorporate these measures in future. Finally, it is worth noting that data collection for this study commenced in academic year 21-22. Whilst there were no COVID-19 related government-imposed restrictions at this time, it is possible that the global pandemic had lasting impacts on health awareness and health behaviours across the population.

## Conclusion

The current study used Latent Profile Transition Analysis to identify characteristics of UK university students at risk of developing adverse markers of health and related behaviours across one year at university. Results indicated that the majority of students were represented in poorer health-related profiles and remain within these profiles throughout the academic year. Of these students, women and TGD students, or students in their second year at university are at greater risk of transitioning to negative health profiles which could also be detrimental to academic performance. University stakeholders should therefore direct funding and resources to developing novel health-based interventions targeted at these at-risk groups to ensure universities are an inclusive, diverse environment within which the health and well-being of all students is considered and supported.

## Data Availability

Data related to the latent transition performed in this study can be found at 10.5281/zenodo.14284040.
